# Toxicity Syndromes, Patient-Related Clinical Indicator of Toxicity Burden Induced by Intensive Triplet Chemotherapy-Based Regimens in Gastrointestinal Cancers With Metastatic Disease

**DOI:** 10.3389/fonc.2020.00172

**Published:** 2020-02-20

**Authors:** Gemma Bruera, Enrico Ricevuto

**Affiliations:** ^1^Oncology Territorial Care, S. Salvatore Hospital, Oncology Network ASL1 Abruzzo, University of L'Aquila, L'Aquila, Italy; ^2^Department of Biotechnological and Applied Clinical Sciences, University of L'Aquila, L'Aquila, Italy

**Keywords:** individual limiting toxicity syndromes, intensive first line, metastatic gastrointestinal cancer, real life, timed-flat-infusion 5-fluorouracil administration, triplet chemotherapy-based regimens, weekly alternating schedule

## Abstract

**Background:** Cancer treatments induce symptoms/signs superimposing on individual patient's clinical status, determining heterogenous toxicity syndromes (TS). We reviewed intensive first line triplet chemotherapy-based regimens in metastatic gastro-intestinal cancers (mGI), based on FIr/FOx schedule, fluorouracil and weekly alternating irinotecan/oxaliplatin, to point out limiting TS (LTS) relevance.

**Methods:** Metastatic colo-rectal (mCRC), pancreatic ductal adenocarcinoma (mPDAC), gastric carcinoma (mGC) patients were enrolled by careful decision-making including age, performance status (PS), and comorbidity status in real life phase II studies: FIr-B/FOx adding bevacizumab (B) overall, FIr-C/FOx-C adding cetuximab (C) in *KRAS*/*NRAS* wild-type mCRC; FIr/FOx in mPDAC; FD/FOx adding docetaxel (D) in mGC. Toxicity, individual LTS, LT alone (LTS-single site, LTS-ss) or associated to other limiting/G2 toxicities (LTS-multiple sites, LTS-ms) were evaluated, compared by chi-square test. In FIr-C/FOx-C, 5-fluorouracil/irinotecan pharmacogenomic biomarkers, 5-fluorouracil degradation rate (5-FUDR), SNPs *ABCB1, CYP3A4, DYPD, UGT1A1* were evaluated, related with LTS.

**Results:** FIr-B/FOx, FIr-C/FOx-C in mCRC, FIr/FOx in mPDAC, FD/FOx in mGC, showed activity, efficacy, toxicities similar to reported triplet regimens. LTS: mCRC FIr-B/FOx 44%, LTS-ms 24%, LTS-ss 20%, in young-elderly 46%, LTS-ms significantly increased vs. LTS-ss; FIr-C/FOx-C 65.5%, significantly increased LTS-ms vs. LTS-ss, in young-elderly 83%; mPDAC FIr/FOx 27.5%, mostly LTS-ms, in young-elderly 38.4% all LTS-ms; mGC FD/FOx 30%, all LTS-ms, in young-elderly 25%. Reduced FUDR, SNPs CYP3A4, UGT1A1, >1 positive pharmacogenomic biomarkers were prevalent in patients with gastrointestinal LTS.

**Conclusions:** LTS is an innovative clinical parameter of toxicity burden, differential treatment-related TS in individual patient. LTS can evaluate pharmacogenomic biomarkers predictive relevance to select mGI patients fit for intensive treatments, at risk of limiting gastrointestinal toxicity.

**Trial Registrations:** The trials were registered at Osservatorio Nazionale sulla Sperimentazione Clinica dei Medicinali (OsSC) Agenzia Italiana del Farmaco (AIFA) Numero EudraCT 2007-004946-34, and Osservatorio Nazionale sulla Sperimentazione Clinica dei Medicinali (OsSC) Agenzia Italiana del Farmaco (AIFA) Numero EudraCT 2009- 016793-32.

## Introduction

### The Need of Patient-Related Clinical Indicator of Toxicity Burden

Addition of more drugs in a chemotherapy combination requires the design of proper schedule and doses, to provide the balance between projected/received (>80%) dose intensity (DI) and treatment-related toxicity (1). This clinical balance is even more challenging to realize for intensive triplet chemotherapy-based regimens that demonstrated to increase clinical outcome in fit metastatic gastro-intestinal (mGI) cancer patients (1–18). Clinical status of the individual patient, also depending from metastatic tumor extension, is the most important variable justifying differential toxicity in individual patients. Thus, patients treated with triplet chemotherapy-based regimens should be enrolled by careful decision-making including age, performance status (PS), and comorbidity status (19).

The conventional evaluation of toxicity of cancer treatments depends upon grading of the clinical relevance of each toxicity symptom and sign, by evaluation of the type, prevalence and intensity of toxicity, more specifically of limiting (G3-4) toxicity, related to the administered treatment regimen, and determined by National Cancer Institute Common Toxicity Criteria (NCI-CTC, version 4.0). Thus, safety profile and toxicities induced by cancer treatments are conventionally defined, according to the number of administered cycles and treated patients, as severe (G3-4), moderate (G2), mild (G1), or absent. This evaluation does not individually describe the toxicity burden experienced in a single patient, defining a spectrum of toxicity syndromes (TS). To better evaluate individual toxicity of intensive treatments, we introduced the concept of Limiting Toxicity Syndromes (LTS), consisting of at least a limiting toxicity alone (LTS-single site, LTS-ss) or associated to other limiting or G2-3 non-limiting toxicities (LTS-multiple sites, LTS-ms) (2, 4, 11, 14, 20). More, symptoms and signs induced by cancer medical treatments add up to clinical general and cancer-related status of the individual cancer patient determining heterogenous TS. A clinical parameter of patient-related toxicity, integrated with conventional treatment-related toxicity evaluation, represents an unmet need in clinical oncology, to verify the occurrence of the spectrum of different toxicities in individual patients, and in a cohort of patients at differential intensity.

We proposed the analysis of TS in different studies developing intensive triplet chemotherapy-based regimens in gastro-intestinal cancer patients with metastatic disease (2, 4, 11, 14, 20), to evaluate the clinical relevance of the integration of patient-related to conventional treatment-related toxicity, to more properly weigh toxicity with treatment-related activity and clinical outcome, and contributing to better address selection of patients suitable for intensive medical treatments. To this aim, LTS represent an innovative clinical parameter of patient-related toxicity burden, indicating global and individual toxicity, consisting of a differential spectrum and intensities of TS, related to administered treatment, according to clinical patient (age, performance status, comorbidity status), and metastatic extension.

### The Model: Intensive First Line Triplet Chemotherapy-Based Regimens in Metastatic Gastrointestinal Cancers

We previously developed doublet chemotherapy schedule of 12 h (10 p.m. to 10 a.m.) timed-flat infusion (TFI) 5-FU (21), associated to irinotecan (CPT-11), safely administered at high 5-FU/DI without leucovorin addiction, with a good tolerability profile, and with activity and efficacy equivalent to reported doublet regimens in metastatic colorectal cancer (mCRC). Then, we designed FIr/FOx triplet chemotherapy schedule by splitting weekly TFI/5-FU (22), and weekly alternating CPT-11 and oxaliplatin (OXP) at DIs 1800, 80, and 40 mg/m^2^/w, respectively, and reported objective response rate (ORR) 66.7%, progression-free survival (PFS) 12 months, and overall survival (OS) 20 months as first line mCRC treatment. Thus, we developed intensive first line regimens, based on FIr/FOx triplet chemotherapy schedule: FIr-B/FOx (2) and FIr-C/FOx-C (4), respectively adding bevacizumab (B) or cetuximab (C), in mCRC; FIr/FOx in metastatic pancreatic ductal adenocarcinoma (mPDAC) (11); FD/FOx in metastatic gastric cancer (mGC), including docetaxel (D) in the same schedule (14).

## Materials and Methods

Patients treated with triplet chemotherapy-based regimens were enrolled by decision-making process including age, performance status (PS), and comorbidity status evaluated by Cumulative Illness Rating Scale (CIRS) (19). CIRS stage was defined as: primary, absent or mild grade comorbidities, and independent Instrumental Activity of Daily Living (IADL); intermediate, <3 mild or moderate grade comorbidities, and dependent or independent IADL; secondary, more than three comorbidities or a severe comorbidity, with or without dependent IADL. Patients with primary or intermediate CIRS stage were enrolled in intensive first line treatment regimens.

Proposed regimens were approved by Agenzia Italiana del Farmaco for administration *in label* for treatment in Italian public hospitals, and published in Gazzetta Ufficiale Repubblica Italiana (“Elenco dei Medicinali erogabili a totale carico del Servizio Sanitario Nazionale,” Gazzetta Ufficiale Repubblica Italiana N.1, 2 Gennaio 2009). MCRC clinical trials were approved by Local Ethical Committee (Comitato Etico, Azienda Sanitaria Locale n.4 L'Aquila, Regione Abruzzo, Italia), and conducted in accordance with Declaration of Helsinki. PDAC study was approved by the Regional Review Board (Regione Abruzzo, Italia, according to D.G.R. n.489, 25/05/2007). All patients provided written, informed consent concerning the proposed treatment, and biological evaluations.

**Figure 1:**
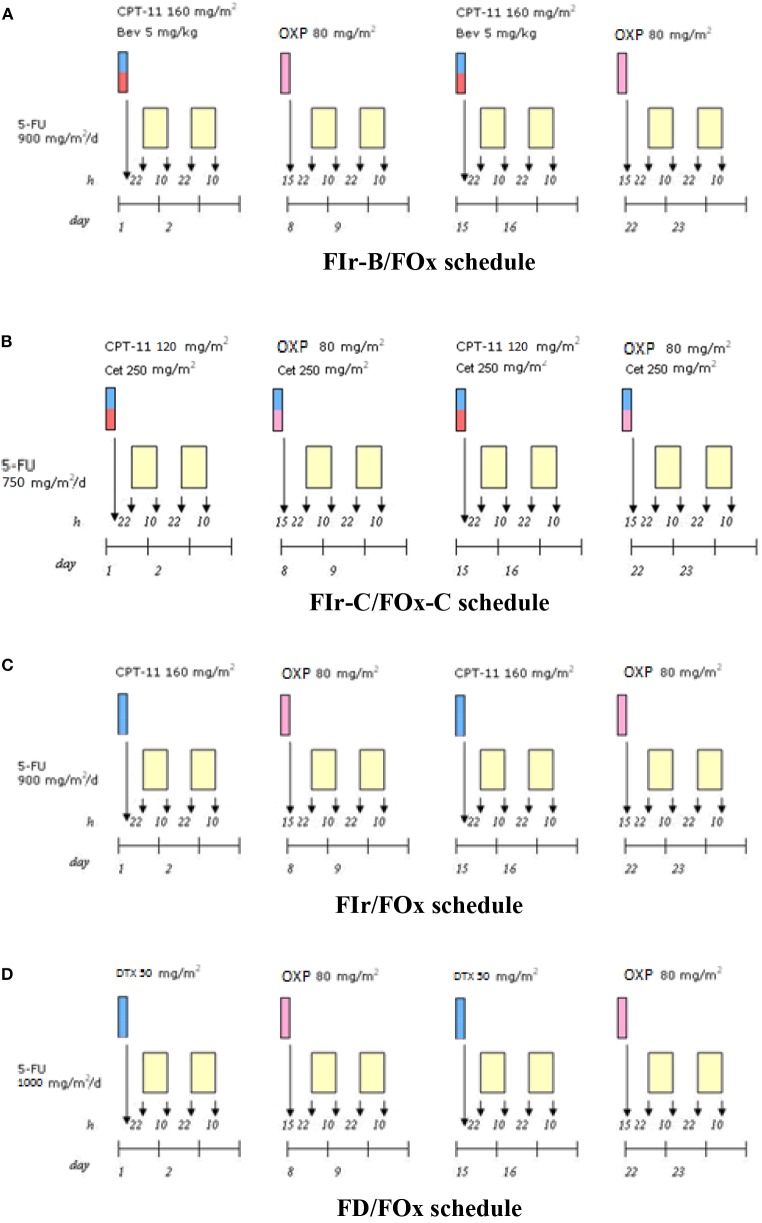
Triplet chemotherapy-based schedules. **(A)** FIr-B/FOx schedule in metastatic colorectal cancer. **(B)** FIr-C/FOx-C schedule in metastatic colorectal cancer. **(C)** FIr/FOx in metastatic pancreatic ductal adenocarcinoma. **(D)** FD/FOx in metastatic gastric cancer.

Figure 1 shows recommended triplet chemotherapy-based regimens developed as first line treatment in mGI cancers. In mCRC, FIr-B/FOx schedule (2): weekly TFI/5-FU 900 mg/m^2^, days 1–2, 8–9, 15–16, 22–23; CPT-11 160 mg/m^2^ days 1,15; OXP 80 mg/m^2^, days 8, 22; every 4 weeks; B 5 mg/kg, days 1,15. In *KRAS*/*NRAS* wild-type mCRC, FIr-C/FOx-C (Figure 1B) (4) weekly C loading dose 400 mg/m^2^, followed by 250 mg/m^2^, was added to triplet FIr/FOx schedule; in subsequent dose-finding steps, 5-FU and CPT-11 were recommended at doses 750 and 120 mg/m^2^, respectively. In mPDAC, FIr/FOx schedule (Figure 1C) (11): TFI/5-FU 900 mg/m^2^/die weekly; CPT-11 160 mg/m^2^, days 1 and 15; OXP 80 mg/m^2^, days 8 and 22. Drug's doses were modulated in patients reporting PS 2, and/or ≥75 years, secondary CIRS stage, and/or liver laboratory tests upper normal limit (≥ G2 hypertransaminasemy at baseline). In mGC, FD/FOx (Figure 1D) (14): TFI/5-FU 1000 mg/m^2^/die weekly; D 50 mg/m^2^ days 1, 15; OXP 80 mg/m^2^ days 8, 22; every 4 weeks.

Conventional toxicity analysis was registered by National Cancer Institute Common Toxicity Criteria (NCI-CTC, version 4.0). To better evaluate toxicity of intensive treatments in the individual patient, LTS, consisting of at least a limiting toxicity alone (LTS-ss) or associated to other limiting or G2-3 non-limiting toxicities (LTS-ms) were evaluated (2, 4, 11, 14, 20). The rates of LTS-ms and LTS-ss were compared by Chi-square test (23). More, in FIr-C/FOx-C study, exploratory analysis of 5-FU/CPT-11 pharmacogenomic biomarkers, specifically 5-FU degradation rate (5-FUDR), defining reduced metabolizers if <1.2 ng/mL/10^6^ cells/min, and Single Nucleotide Polimorphisms (SNPs) *ABCB1* (C3435T, C1236T), *CYP3A4* (1B, 53), *DYPD1* (IVS14+1, A166G), UGT1A1 (28) were preliminarily related with the occurrence of LTS (4, 24, 25).

## Results

### Clinical Outcome and Toxicity

In mCRC patients, FIr-B/FOx and FIr-C/FOx-C were recommended at different 5-FU/CPT-11 doses, 900/160 and 750/120 mg/m^2^, respectively (Table 1) (2, 4). In overall and *KRAS*/*NRAS* wild-type mCRC patients treated with FIr-B/FOx, and FIr-C/FOx-C, respectively, median received DI (rDI) per cycle for all the drugs at the recommended doses were ≥80% (2, 4). Reported ORR were, respectively, 82 and 78%, PFS equivalently 12 months, OS 28 and 23 months. In young-elderly (yE) mCRC patients (≥65 <75 years) treated with FIr-B/FOx, ORR was 79%, median PFS 11 months, median OS 21 months (20). In yE mCRC patients treated with FIr-C/FOx-C, median rDIs per cycle were <80%: 5-FU 71% (1066 mg/m^2^/w); CPT-11 76% (45.5 mg/m^2^/w); OXP 66% (26.5 mg/m^2^/w); C 71% (178.5 mg/m^2^/w); yE patients showed significantly worse OS compared to non-elderly (*P* 0.045) (4). Overall, FIr-C/FOx-C treatment was discontinued due to LT in 48% patients, prevalently due to diarrhea. Prevalent G3-4 toxicities by patients treated with FIr-B/FOx and FIr-C/FOx-C at recommended doses were, respectively: diarrhea 28 and 23%, asthenia 6 and 15%, neutropenia 10% and none, vomiting 4 and 8%, mucositis 6% and none, hypertension 2% and none, hypokalemia 2% and none, hypertransaminasemy 4 and 8% (2, 4).

**Table 1:** Treatment regimens, clinical outcome, and safety profile.

	**Metastatic** **colorectal** **cancer**				**Metastatic pancreatic ductal adenocarcinoma**		**Metastatic gastric cancer**	
	**DI/cycle** **mg/m**^****2****^**/w or mg/kg/w**		**DI/cycle** **mg/m**^****2****^**/w**		**DI/cycle** **mg/m**^****2****^**/w**		**DI/cycle** **mg/m**^****2****^**/w**	
	**FIr-B/FOx**		**FIr-C/FOx-C**		**FIr/FOx**		**FD/FOx**	
**No. patients**	**50**		**29**		**29**		**10**	
**Drugs**	**Projected dose** **mg/m^**2**^ or mg/Kg**	**rDI (%)**	**Projected dose** **mg/m^**2**^**	**rDI (%)**	**Projected dose** **mg/m^**2**^**	**rDI (%)**	**Projected dose** **mg/m^**2**^**	**rDI (%)**
5-Fluorouracil	1800	82.6	1500	85.3	1800	70.4	2000	82.9
Irinotecan	160	83	120	93.3	160	70	-	-
Oxaliplatin	80	80	80	80	80	72.5	80	83.8
Bevacizumab	5	84	-	-	-	-	-	-
Cetuximab	-	-	250	80	-	-	-	-
Docetaxel	-	-	-	-	-	-	50	91
Clinical outcomes								
ORR (%)	82		78		53		60	
PFS (months)	12		12		4		6	
OS (months)	28		23		11		17	
**Limiting toxicities (%)**								
Diarrhea	28		23		17		-	
Nausea	6		-		3		-	
Vomiting	4		8		3		-	
Hypoalbuminemia	-		-		3		10	
Mucositis	6		-		6		10	
Asthenia	6		15		14		20	
Ipokaliemia	2		-		7		-	
Hypertransaminasemia	4		8		7		-	
Neutropenia	10		-		17		50	
Thrombocytopenia	-		-		3		-	
Anemia	-		-		3		-	

*DI, dose-intensity; rDI, received dose-intensity; ORR, objective response rate; PFS, progression-free survival; OS, overall survival*.

In mPDAC patients, FIr/FOx showed ORR 53%, PFS 4 months, OS 11 months; among yE/old-elderly patients, median PFS 4 months, and median OS 5 months (11). Elderly patients did not showed significantly worse PFS and OS compared to non-elderly patients. Patients with PS 2 showed significantly worse OS compared with PS 0-1 patients (*P* = 0.022). In mPDAC patients treated with FIr/FOx, median rDI per cycle were: 5-FU 70.4% (1268.5 mg/m^2^/w); CPT-11 70% (56 mg/m^2^/w); OXP 72.5% (29 mg/m^2^/w). In yE, 5-FU 83.3%, CPT-11 80%, OXP 85%. Overall, 17% discontinued FIr/FOx treatment due to LT. Limiting cumulative G3-5 toxicities were: diarrhea 17%, asthenia 14%, neutropenia 17%, mucositis 6%, hypokaliemia 7%, hypertransaminasemia 7%, nausea/vomiting, hypoalbuminemia, anemia, thrombocytopenia 3%, respectively. One case of toxic death (3%) was observed.

In mGC patients, FD/FOx at recommended 5-FU 1000 mg/m^2^/day, D 50 mg/m^2^ and OXP 80 mg/m^2^, showed ORR 60%, PFS 6 months, OS 17 months (14). Median rDI were: 5-FU 82.9% (1657.5 mg/m^2^/week); D 91% (22.75 mg/m^2^/week); OXP 83.8% (33.5 mg/m^2^/week). Cumulative G3-4 toxicities were represented by: asthenia 20%, neutropenia 50%, leucopenia 20%, mucositis and hypoalbuminemia 10%.

### Evaluation of Individual Toxicity Syndromes (TS) and Relationship With Companion Pharmacogenomic Analysis

In *KRAS*/*NRAS* wild-type mCRC patients treated with FIr-B/FOx, overall LTS were observed in 22 patients (44%) (Table 2): LTS-ms 12 (24%); LTS-ss 10 (20%) (2). LTS-ms characterized by ≥2 LT 2 (4%); LT associated to other, at least G2, non-limiting toxicities 10 (20%). In yE mCRC treated with FIr-B/FOx, LTS were observed in 13 out of 28 patients (46%): LTS-ms, 11 (39%); LTS-ss, 2 (7%) (20). LTS-ms were characterized by: ≥2 LTs, 2 (7%), LT associated to G2-3 toxicities, 9 (32%). In elderly patients compared to non-elderly, LTS were significantly represented by LTS-ms vs. LTS-ss (chi square 3.832, *P* 0.05). LTS were prevalently characterized by G2-3 diarrhea, 9 patients (69.2%), 8 LTS-ms and 1 LTS-ss. The 2 LTS-ms with double LT were observed in yE patients. The 10 LTS-ms, defined by LT added to other, at least G2, non-limiting toxicities, were prevalently determined by G2-3 diarrhea (90%), plus G2-3 nausea/vomiting (70%). The 10 LTS-ss were prevalently characterized by G3 diarrhea (50%), G3 asthenia, hypertension, hypertransaminasemy, neutropenia, thrombocytopenia 10%, respectively.

**Table 2:** Individual Toxicity Syndromes: overall LTS, LTS-ms and LTS-ss, according to triplet chemotherapy-based regimen.

	**Metastatic colorectal cancer**				**Metastatic pancreatic ductal adenocarcinoma**		**Metastatic gastric cancer**	
	**FIr-B/FOx**		**FIr-C/FOx-C**		**FIr/FOx**		**FD/FOx**	
	**N**.	**%**	**N**.	**%**	**N**.	**%**	**N**.	**%**
Overall patients	50		29		29		10	
Toxicity syndromes	22	44	19	65.5	8	27.5	3	30
LTS-ms	12	24	17	59	7	24.1	3	30
LTS-ss	10	20	2	7	1	3.4	-	-
Young-elderly patients	28	42	6	24	13	34.4	4	40
Toxicity syndromes	13	46	5	83	5	38.4	1	25
LTS-ms	11	39	4	67	5	38.4	1	25
LTS-ss	2	7	1	17	-	-	-	-

LTS, limiting toxicity syndromes; LTS-ms, LTS multiple sites; LTS-ss, LTS single site.

In *KRAS*/*NRAS* wild-type mCRC patients treated with FIr-C/FOx-C, LTS were observed in 19 patients (65.5%), 5 out of 6 yE (83%): LTS-ms 17 (59%), LTS-ss 2 (7%) (4). LTS were significantly represented by LTS-ms vs. LTS-ss (chi square 7.703, *P* 0.006). LTS-ms characterized by ≥2 LT 7 (24%); LT associated to other toxicities 10 (34%). LTS were prevalently characterized by G3-4 diarrhea and G3 asthenia associated to other toxicities.

In mPDAC patients treated with FIr/FOx, overall LTS were 8 (27.5%); 5 out of 13 yE/old-elderly (38.4%); LTS-ms 7 (24.1%), LTS-ss 1 (3.4%) (11). LTS-ms > 2 LTs 1 (3.4%); LT associated to non-limiting toxicities 6 (20.6%). LTS were not significantly represented by LTS-ms compared to LTS-ss. In mGC patients treated with FD/FOx, overall LTS were observed in 3 patients (30%), all LTS-ms: 1 out of 4 yE (25%); 2 out of 6 non-elderly (33.3%) (14).

Furthermore, the analysis of SNPs represented an exploratory analysis in colorectal cancer patients treated with intensive triplet chemotherapy plus cetuximab, according to FIr-C/FOx-C schedule, to better evaluate the safety profile, particularly gastrointestinal. Patients who reported gastrointestinal LTS, prevalently showed reduced fluorouracil degradation rate (FUDR), Single Nucleotide Polimorphisms (SNP) *CYP3A4, UGT1A1*, and >1 positive pharmacogenomic biomarkers. Specifically, the exploratory analysis of 5-FUDR reduction and *ABCB1, CYP3A4, DYPD, UGT1A1* SNPs evaluated in 14 *KRAS*/*NRAS* wild-type mCRC patients (48.3%) treated with FIr-C/FOx-C (4, 24, 25), and compared with LTS occurrence in 47.4% of patients who showed LTS, reported prevalent 5-FUDR reduction, and *CYP3A4, UGT1A1* SNPs in patients with gastrointestinal LTS; 78% of mCRC patients with LTS showed >1 pharmacogenomic alterations, including reduced 5-FUDR, *CYP3A4* and/or *UGT1A1* SNPs (range 1–3).

## Discussion: Clinical Relevance of the Integration of TS in the Evaluation of Toxicity

Over the past 10 years, we developed intensive triplet chemotherapy-based regimens in fit mGI cancer patients (2, 4, 11, 14), showing activity and clinical outcomes similar to that reported (3, 5–10, 12, 13, 15–18), and characterized by FIr/FOx schedule design consisting of weekly administration of 5-FU and weekly alternated administration of two other drugs, such as CPT-11 or OXP (22), in order to recommend schedules providing an adequate balance between received DI ≥ 80% and treatment-related toxicity (1). Clinical status of the individual patient, also depending from metastatic tumor extension, is the most important variable justifying differential toxicity in individual patients. Thus, patients treated with triplet chemotherapy-based regimens should be enrolled by careful decision-making including age, PS, and comorbidity status (19, 20, 26, 27).

To this aim, FIr-B/FOx (2) as first line treatment of mCRC patients gained equivalent efficacy than that reported with triplet schedules, such as FOLFOXIRI/BEV (2, 3, 28), and demonstrated a good tolerability profile (1, 29), with lower G3-4 neutropenia, also in yE mCRC patients (20, 27). C addiction according to FIr-C/FOx-C schedule in *KRAS*/*NRAS* wild-type mCRC (4) met the projected high activity, as different other schedules of C addition to triplet chemotherapy, chrono-IFLO, ERBIRINOX, FOLFOXIRI (5–8), or panitumumab addiction to modified FOLFOXIRI (9, 10), even if individual toxicity profile limited the wide use of intensive schedules associating triplet chemotherapy and anti-EGFR targeted agents in clinical practice, thus requiring pharmacogenomic analysis to more properly select fit mCRC patients (4). FIr/FOx schedule in mPDAC patients may increase activity and efficacy, as previously reported in mCRC patients (11), even if it required modulation of doses reducing median rDI <80%, and PS 2 may affect significantly worse OS. FD/FOx schedule in mGC patients was feasible at median rDI > 80%, and showed equivalent efficacy as D associated to cisplatin/5-FU (16, 17), with good tolerability (14). In randomized studies, D addiction to cisplatin/5-FU-based triplet chemotherapy regimen (16, 17), showed significantly increased toxicity, that limited the expected efficacy of the triplet regimen.

The conventional way to measure toxicity of cancer treatments is represented by the description of each type of toxicity according to National Cancer Institute Common Toxicity Criteria (NCI-CTC, version 4.0), to obtain the description of cumulative and prevalent limiting (G3-G4), moderate (G2), mild (G1), or absent toxicities directly determined by the cancer treatment, according to the number of administered cycles and treated patients. This way does not really represent the clinical burden of toxicity in the individual patient, nor define prevalent individual TS affecting a patient population equivalently treated and their variability. Thus, in reported intensive first line triplet chemotherapy-based regimens developed in mGI cancer patients, we added the description of individual TS, and specifically of LTS, to describe cumulative and individual toxicity, that include differential spectrum and intensities of TS, depending from medical treatment and patients' individual clinical conditions.

The integration of the evaluation of LTS to the conventional treatment-related toxicity, contributed a patient-related clinical indicator of toxicity burden, providing a global evaluation of patient-related limiting toxicity (2, 4, 11, 14, 20): in mCRC patients treated with FIr-B/FOx or FIr-C/FOx-C, LTS were 44 and 65.5%, respectively; in mPDAC patients treated with FIr/FOx, 27.5%; in mGC patients treated with FD/FOx, 30%. LTS also provided a classification of LTS according to the spectrum and intensity of toxicities: in mCRC patients treated with FIr-B/FOx, LTS-ms were equivalent to LTS-ss; in mCRC patients treated with FIr-C/FOx-C, LTS-ms were significantly prevalent 59% and frequently characterized by LTS with ≥2 LT 24%, and they were also prevalent in mPDAC patients treated with FIr/FOx and in mGC patients treated with FD/FOx.

In yE patients, LTS evaluation showed the differential tolerability of intensive triplet chemotherapy based regimens: in mCRC, FIr-B/FOx LTS 46%, but significantly prevalent LTS-ms (20, 27); FIr-C/FOx-C LTS 83%, pointing elderly status ≥65 years as an exclusion criteria for intensive regimens adding triplet chemotherapy to anti-EGFR. In mPDAC patients, FIr/FOx LTS 38.4%; in mGC patients treated with FD/FOx, LTS 25%. Specifically, LTS also provided an indicator of individual, patient-related toxicity useful for proper treatment and care in clinical practice.

Furthermore, even if performed in only 14 patients (48.3% treated) and in 9 who showed LTS (47.4%), exploratory data of pharmacogenomic biomarkers compared with LTS occurrence in *KRAS*/*NRAS* wild-type MCRC patients treated with FIr-C/FOx-C showed that reduced 5-FUDR, and *CYP3A4* and *UGT1A1* SNPs may predict individual LTS occurrence, particularly at recommended doses, specifically gastrointestinal LTS (4). Thus, LTS can represent the innovative and proper indicator to whom relate pharmacogenomic analysis and it may guide proper selection of patients suitable for intensive regimens adding triplet chemotherapy and anti-EGFR drug, or to modulate intensive triplet chemotherapy-based regimens.

Thus, LTS meets the need of an innovative clinical parameter of patient-related toxicity burden, to measure personalized safety of intensive first line triplet chemotherapy-based regimens proposed in mGI, also associated to targeted agents (B or C). Its clinical relevance should be prospectively evaluated as a model in clinical practice. The integration of LTS and conventional toxicity evaluations may help proper selection of patients fit for intensive first line medical treatments in mGI cancers, to more properly weigh toxicity analysis with activity and clinical outcome and its contribution to address selection of patients suitable for intensive triplet chemotherapy-based regimens. The equivalent, integrated evaluation and monitoring of individual safety by LTS, can help properly select first line intensive medical treatment, and safely administer and manage an intensive first line regimen, that could guarantee increased clinical outcomes and good safety profile in real life.

In the era of precision oncology, integrating molecular characterization of cancer affecting the individual patient to specifically address targeted treatments, such as in mCRC (30–32), the addition of LTS could integrate the description of cumulative toxicities, toward a precision toxicity evaluation, even to better evaluate innovative drugs as intensive combinations of multiple drugs, favoring the dissemination of innovative treatments in clinical practice.

Analysis of TS could be integrated in the therapeutic pathway of cancer patients in clinical practice and in clinical studies to globally evaluate tolerability of cancer treatments. It could be, also, particularly useful for a more proper evaluation of tolerability in the therapeutic pathway of individual cancer patients unfit for standard treatments, due to elderly status and/or their clinical status, or unfit for intensive regimens (33, 34), to optimize simultaneous care of cancer patients (35), and in patients treated with innovative non-infusional targeted-drugs, as well as in adjuvant treatments of early cancers, to even more properly weigh the balance between adjunctive efficacy of cancer treatment and its safety in potentially curable patients.

More, in intensive regimens such as FIr-C/FOx-C, characterized by high discontinuation rate of treatment, highly prevalent (>50%) LTS and heterogeneity of LT, LTS may help directly relate individual patient- and drugs-related toxicity with pharmacogenomics biomarkers referred to the individual genetic identity of the cancer patient.

## Conclusion

TS, specifically LTS, represents an innovative clinical parameter of cumulative and individual patient-related toxicity burden, defining differential spectrum and intensities of treatment-related TS, depending from the clinical status of the individual cancer patient, particularly according to elderly, and PS.

## Data Availability Statement

The raw data supporting the conclusions of this article will be made available by the authors, without undue reservation, to any qualified researcher.

## Ethics Statement

Proposed regimens were approved by Agenzia Italiana del Farmaco for administration in label for treatment in Italian public hospitals, and published in Gazzetta Ufficiale Repubblica Italiana (Elenco dei Medicinali erogabili a totale carico del Servizio Sanitario Nazionale, Gazzetta Ufficiale Repubblica Italiana N.1, 2 Gennaio 2009). MCRC clinical trials were approved by Local Ethical Committee (Comitato Etico, Azienda Sanitaria Locale n.4 L'Aquila, Regione Abruzzo, Italia), and conducted in accordance with Declaration of Helsinki. PDAC study was approved by the Regional Review Board (Regione Abruzzo, Italia, according to D.G.R. n.489, 25/05/2007). All patients provided written, informed consent concerning the proposed treatment, and biological evaluations.

## Author Contributions

GB contributed in conceptualization, data curation, formal analysis, investigation, methodology, project administration, validation, and writing original draft. ER contributed in conceptualization, data curation, formal analysis, investigation, methodology, project administration, supervision, validation, and writing—review original draft.

### Conflict of Interest

The authors declare that the research was conducted in the absence of any commercial or financial relationships that could be construed as a potential conflict of interest.

## Abbreviations

B: bevacizumab
C: cetuximab
CIRS: Cumulative Illness Rating Scale
CPT-11: irinotecan
D: docetaxel
DI: dose-intensity
IADL: Instrumental Activity of Daily Living
LTS: limiting toxicities syndromes
LTS-ms: limiting toxicity syndrome multiple sites
LTS-ss: limiting toxicity syndrome single site
mCRC: metastatic colorectal cancer
mGC: metastatic gastric cancer
mGI: metastatic gastro-intestinal
mPDAC: metastatic pancreatic ductal adenocarcinoma
NCI-CTC: National Cancer Institute—Common Toxicity Criteria
ORR: objective response rate
OS: overall survival
OXP: oxaliplatin
PFS: progression-free survival
PS: performance status
rDI: received dose-intensity
SNP: Single Nucleotide Polimorphisms
TFI: timed-flat-infusion
TS: toxicities syndromes
yE: young-elderly
5-FU: 5-fluorouracil
5-FUDR: 5-fluorouracil degradation rate.
